# What *Babesia microti* Is Now

**DOI:** 10.3390/pathogens10091168

**Published:** 2021-09-10

**Authors:** Heidi K. Goethert

**Affiliations:** Cummings School of Veterinary Medicine, Tufts University, Grafton, MA 01536, USA; Heidi.goethert@tufts.edu

**Keywords:** *Babesia microti*, *Babesia*, diversity, phylogenetic analysis

## Abstract

Parasites from diverse hosts morphologically identified as *Babesia microti* have previously been shown to belong to a paraphyletic species complex. With a growing number of reports of *B. microti*-like parasites from across the world, this paper seeks to report on the current knowledge of the diversity of this species complex. Phylogenetic analysis of 18S rDNA sequences obtained from GenBank shows that the diversity of the *B. microti* species complex has markedly increased and now encompasses at least five distinct clades. This cryptic diversity calls into question much of our current knowledge of the life cycle of these parasites, as many biological studies were conducted before DNA sequencing technology was available. In many cases, it is uncertain which *B. microti*-like parasite was studied because parasites from different clades may occur sympatrically and even share the same host. Progress can only be made if future studies are conducted with careful attention to parasite identification and PCR primer specificity.

*Babesia microti* has historically been identified by the intra-erythrocytic morphology of the parasites, typically appearing in infected cells as small (1–2.0 µm in diameter) basket-shaped rings with extended chromatin. Relying solely on this morphology for identification, parasites from diverse hosts, such as shrews, mice, rats, raccoons, and dogs, are all referred to as *B. microti* or *B. microti*-like [1,2,3,4]. In 2003, a phylogenetic study was published demonstrating that such parasites are not a single organism. Sequences from both the 18S ribosomal and the beta-tubulin genes were paraphyletic, demonstrating that *B. microti* is, in fact, a species complex comprised of three genetically distinct clades [5]. Furthermore, parasites from only one of the clades are responsible for most human babesiosis cases. Indeed, cryptic diversity is common among parasites with few morphological traits that can be used for differentiation [6]. In the intervening 20 years, there has been a growing number of reports of *B. microti*-like parasites from diverse hosts worldwide. It is now clear that this species complex is even more diverse than originally described. This paper seeks to report the current knowledge of the species diversity and clarify, once again, what *Babesia microti* is.

To this aim, all the 18S ribosomal DNA sequences >800 bp currently in GenBank that are either named *Babesia microti* in the database or appear on a Blast search with a sequence similarity of >95% to the *B. microti* human strains from the United States were downloaded. Although the 18S gene may not be the best target for describing diversity because of its highly conserved nature, it is the only gene that is reliably sequenced from the majority of studies. Limiting this analysis to large pieces of the gene maximizes the amount of diversity obtained from this conserved gene. From the large list of sequences available, a sample was chosen that attempted to encompass the genetic diversity of the entire database while removing large numbers of highly similar sequences that make the trees difficult to interpret. Thirty-nine sequences were aligned using Geneious (GenBank numbers are in the figure) and then trimmed so that they were all the same length, corresponding to bases 478–1350 from the Gray strain (GenBank #AY693840). A neighbor-joining tree was constructed with MEGA X [7] using *B. divergens* and *B. leo* as outgroups (Figure 1). This new analysis reveals that the three originally described clades remain, but they are joined by at least 2 additional clades.

Clade 1: Clade 1 has also been referred to as *B. microti sensu stricto* (or the US-type), as the parasites from this clade are arguably the most important because of their public health impact as the major cause of human babesiosis worldwide. This parasite is also the most studied. Clade 1 parasites are remarkably conserved, with virtually identical 18S rDNA sequences described across the globe: North America, Europe, and Asia. Despite this, human cases are only common in the United States. There, people are readily exposed because of the highly anthropophilic tick *Ixodes dammini* (the northern clade of *Ixodes scapularis)* that serves as its main vector [8,9]. In Europe, *B. microti ss* is thought to be maintained by *I. trianguliceps*, a host-specific tick that does not attack people, which would explain the lack of human disease. However, much of the work with *I. trianculiceps* was conducted before molecular methods were available, and it is uncertain whether the parasite under study belonged to this clade or clade 3 (For example see [3,10,11]). To date, no definitive molecular sequencing of field-derived *I. trianguliceps* has shown *B. microti* ss in these ticks. *I. ricinus*, an anthropophilic tick that serves as the vector for Lyme disease, is often sympatric, feeds on similar rodent reservoir hosts, and has been shown to be capable of transmitting piroplasms in the laboratory [12]. Indeed, *B. microti* ss is regularly detected in this vector, as well as *I. persulcatus* in Eurasia [13,14,15]. Therefore, the zoonotic potential should exist throughout the range of these ticks in Europe and Asia. Serosurveys show that tick-exposed people are indeed exposed to the parasite, but few cases of illness have been detected [16,17]. Whether the parasites are less virulent in the rest of the world compared to those in North America or whether physicians fail to diagnosis this disease because of lack of physician awareness and diagnostic capabilities has not been determined [18].

Clade 2: Clade 2 includes *Babesia spp.* that are known to infect carnivores, including raccoons, foxes, and badgers across the world. Also included in this clade is the parasite originally described from sick domestic dogs from Spain, which has been called by many names: *B. microti*-like, *Babesia* c.f. *microti, Theileria annae*, and *Babesia annae* [2,19]. Recently, it has been proposed that this parasite be designated a new species called *Babesia vulpes* [20,21]. The phylogenetic trees published by Baneth et al. in 2015 suggest that they propose the name *B. vulpes* only be applied to the *Babesia* in wild foxes, which also causes disease in domestic dogs, but not to the closely related *Babesia* found in other carnivores. The current phylogenetic analysis clearly shows that other sequences from raccoons and badgers group with *B. vulpes* in a strongly supported clade to the exclusion of the other *B. microti*-like parasites. If the name *B. vulpes* is indeed adopted, it seems unduly confusing to continue to refer to the other carnivore *Babesia* as *B. microti*-like. The vectors for the parasites in this clade have not been definitively established but are likely to be *Ixodid* ticks that feed primarily on carnivores, such as *I. texanus* in North America. In Europe, *I. hexagonus* and *Dermacentor reticulatus* have both been suggested as possible vectors [22,23]. It may also be transmitted by other routes that do not involve a vector, such as direct transmission through bites [24].

Not included within Clade 2 are the sequences from skunks originally described from Massachusetts [6]. The phylogenetic position of these skunk sequences is unstable, as they either cluster with the *B. vulpes* group, with the *B. microti* ss group, or, as is the case in this analysis, separated from both, depending on the type of algorithm used (see [6]). To date, no other similar sequences have been described, despite recent work in the U.S. characterizing small *Babesia* in medium-sized mammals [25,26,27], leaving our knowledge of this parasite limited and their placement among the *B. microti* clade uncertain. It is clear, however, that these piroplasms are distinct from the previously described *Babesia* from skunks, *B. mephitis* [28].

Clade 3: Clade 3 includes *B. microti* similar to the Munich strain that have been primarily detected from voles. These piroplasms occur in Europe and North America but have not been found in Asia. There is distinct separation in this clade between the sequences originating from the two continents. The *Babesia* from this clade are not known to cause human infection. In fact, they have been detected from areas of the US where human babesiosis has not been described and the anthropophilic vector, *I. dammini*, is not present [29,30]. Instead, *I. angustus* is known to occur in these areas, suggesting that this host-specific tick that rarely bites humans is the major vector in North America and the reason that this piroplasm is not known to infect humans. In Europe, the Munich type appears to be present only in areas where *I. trianguliceps* occurs, and it has been suggested that this tick is the primary vector [31]. However, throughout much of its range, either *I. ricinus* or *I. persulcatus* also occur, and as mentioned above, many ecological studies could not discern between the Munich-type and US-type parasites. Rar et al. [32] showed that in an area with sympatric *I. trianguliceps* and *I. persulcatus*, Munich-type *B. microti* was only detected in *I. trianguliceps* [32] and concluded that *I. persulcatus* was not the vector. However, others have detected sequences consistent with the Munich-type in *I. ricinus* [33,34,35], thus leaving the zoonotic potential for this piroplasm and the enzoonotic cycle in nature uncertain.

Clades 4 and 5: Clades 4 and 5 comprise *Babesia* that have only been detected in Asia. Clade 4 includes the Kobe strain from Japan together with sequences derived from China. Clade 5 comprises the Hobetsu and Otsu types, along with other sequences from voles from China. Although often referred to as if they are different parasites, these are actually the same and have 100% sequence similarity in the 18S rDNA gene (only Hobetsu is shown on the tree in Figure 1). None of the parasites from either clade have been detected in Europe or North America, though the US-type occurs sympatrically with both these parasites in Asia. Hobetsu parasites have been found primarily in Japan, with one report in rodents from mainland China [36,37], but Kobe appears to be more widespread in Japan, mainland China, and other parts of southeastern Asia and has been detected in more diverse rodent hosts [38,39,40]. In Japan, parasites from these two clades are often sympatric, but only the Kobe clade has been shown to infect humans. The Hobetsu strain has been detected in I. ovatus, and laboratory studies have confirmed the competence of this vector [37]. However, the vector for the Kobe strain remains undescribed; to date, it has never been detected in field-collected ticks. There is an odd report from a sick domestic cat in South Africa, which appeared to be coinfected with *B. felis* and *B. microti*-like parasites with 100% similarity to the Hobetsu strain [41]. This lone report remains an anomaly, as cats are not otherwise known to become infected with *B. microti*-like parasites, though they do harbor other small *Babesia* that are more closely related to *B. rodhaini*, *B. leo*, and *B. felis* [42].

Finally, there are a number of sequences from GenBank which fall within the *B. microti* species complex but do not group with other previously described parasites to create well-defined clades. The vast majority of these new sequences originate from rodents or ticks collected primarily in China but also Japan. Most remain as single reports or unpublished sequences deposited in GenBank, so little is known about their life cycles or their zoonotic potential. Interestingly, similar sequences were detected in squirrels collected in Japan and macaques from China [43,44]. Further investigations are necessary to characterize these parasites as well as create isolates. In the future, there will likely be 4 additional clades added to the *B. microti* species complex.

As this analysis shows, the parasites that are part of the *B. microti* species complex are a diverse group with unique life cycles. However, the understanding of the ecology of these parasites has been muddled because of the lack of precision in many studies and the confusion between similar parasites. As pointed out above, much of the basic biology of *B. microti*, both in the US and Europe, was conducted before molecular methods were available to distinguish between parasites. It is virtually impossible to know for certain which parasite, Clade 1 *B. microti ss* or Clade 3 Munich-type, was being studied in the older literature (for example, [45,46]), but also in more recent work (for example, [11,47,48]). In the United States in particular, researchers have focused their efforts on *B. microti* ss because of its public health importance there. Indeed, most studies are conducted in areas of the country where human cases are detected and presume the presence of that single parasite. This is likely to be an accurate assumption when surveying ticks. To date, *B. microti* ss is the only *B. microti*-like parasite found in the zoonotic vectors *I. dammini* and *I. scapularis*. Other *Ixodes* ticks that are more host-specific, such as *I. cookei* and *I. angustus*, are rarely studied. Surprisingly few studies in the U.S. have actually sequenced PCR amplicons obtained from wildlife sources to confirm the identity of *B. microti* unless the host is from an area where human babesiosis has not been detected. Unexpected results can arise when performing due diligence to confirm the identity of parasites (see [29,30] and the descriptions of *B. conradae* [49] and *B. duncani* [50]). Therefore, it is imperative at the start of any new study to confirm the identity of parasites by sequencing using a sufficiently informative gene segment (such as [30,51]). Once the specific clade of *B. microti* has been confirmed, it is not necessary to sequence every positive PCR given that the primers used are specific enough to amplify only the intended target. The use of non-specific PCR primers that are capable of amplifying other *B. microti*-like parasites can call into question the conclusions of a paper. Many different primer sets have been used in the literature, and a quick search using PrimerBlast from NCBI is useful to give a reader an estimate of their specificity. This issue becomes even more crucial with the use of real-time PCR, which usually amplifies small pieces of DNA that cannot be confirmed subsequently by sequencing.

It is clear from this analysis that there is much still unknown about the basic biology of the many parasites that make up the *B. microti* species complex. However, progress will only be obtained with well-designed studies that are careful to identify which *B. microti*-like parasite is being studied.

## Figures and Tables

**Figure 1:**
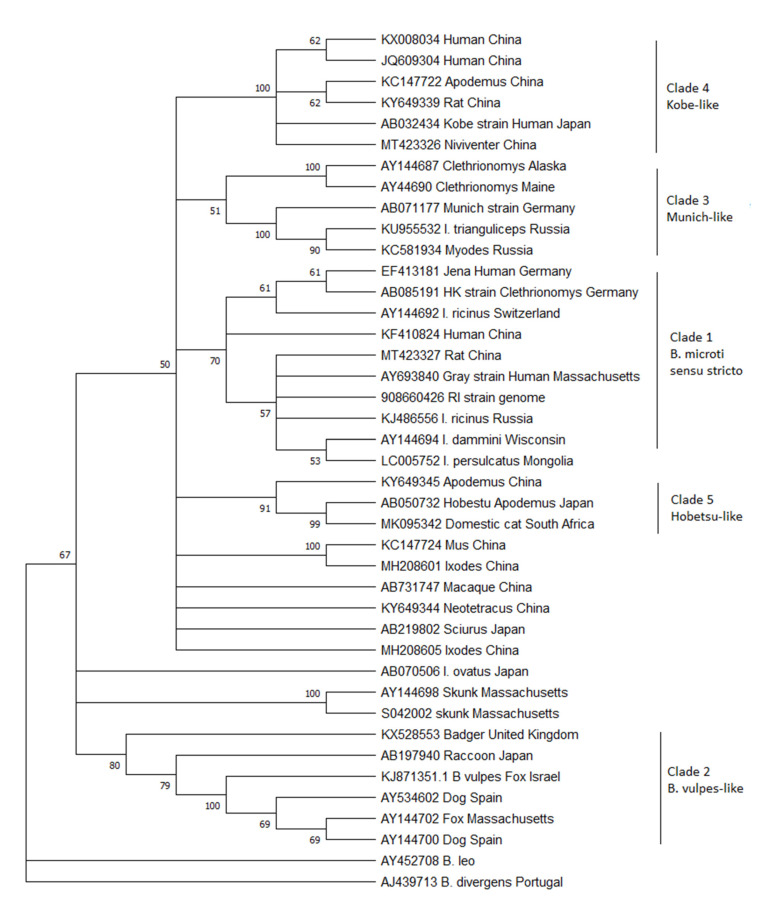
Phylogenetic analysis of the 18S rDNA gene of *B. microti*-like piroplasms. A neighbor-joining tree was constructed using MEGA X with 500 bootstrap replicates. Evolutionary distances were calculated using the Kimera-2 parameter method with *B. divergens* and B. leo as outgroups. Branches with less than 50% bootstrap support were consolidated. GenBank accession numbers are listed on the tree.

## Data Availability

All data used in the study is publicly available from GenBank. Accession numbers are included in the figure.

## References

[1] Spielman A., Etkind P., Piesman J., Ruebush T.K., Juranek D.D., Jacobs M.S. (1981). Reservoir Hosts of Human Babesiosis on Nantucket Island. Am. J. Trop. Med. Hyg..

[2] Camacho A.T., Guitián F.J., Pallas E., Gestal J.J., Olmeda A.S., Goethert H.K., Telford S.R. (2001). Infection of Dogs in North-West Spain with a *Babesia Microti*-like Agent. Vet. Rec..

[3] Young A.S. (1970). Investigations on the Epidemiology of Blood Parasites of Small Mammals with Special Reference to Piroplasms. Ph.D. Thesis.

[4] Healing T.D. (1981). Infections with Blood Parasites in the Small British Rodents Apodemus Sylvaticus, Clethrionomys Glareolus and Microtus Agrestis. Parasitology.

[5] Goethert H.K., Telford III S. (2003). What Is Babesia Microti?. Parasitology.

[6] Perkins S.L., Martinsen E.S., Falk B.G. (2011). Do Molecules Matter More than Morphology? Promises and Pitfalls in Parasites. Parasitology.

[7] Kumar S., Stecher G., Li M., Knyaz C., Tamura K. (2018). MEGA X: Molecular Evolutionary Genetics Analysis across Computing Platforms. Mol. Biol. Evol..

[8] Spielman A. (1976). Human Babesiosis on Nantucket Island: Transmission by Nymphal Ixodes Ticks. Am. J. Trop. Med. Hyg..

[9] Spielman A., Wilson M.L., Levine J.F., Piesman J. (1985). Ecology of *Ixodes Dammini*-Borne Human Babesiosis and Lyme Disease. Ann. Rev. Ent..

[10] Krampitz H.E. (1979). *Babesia Microti*: Morphology, Distribution and Host Relationship in Germany. Zentralbl. Bakteriol. Orig. A.

[11] Randolph S.E. (1991). The Effect of *Babesia Microti* on Feeding and Survival in Its Tick Vector, Ixodes Trianguliceps. Parasitology.

[12] Gray J., von Stedingk L.V., Gürtelschmid M., Granström M. (2002). Transmission Studies of *Babesia Microti* in Ixodes Ricinus Ticks and Gerbils. J. Clin. Microbiol..

[13] Duh D., Petrovec M., Avsic-Zupanc T. (2001). Diversity of *Babesia* Infecting European Sheep Ticks (Ixodes Ricinus). J. Clin. Microbiol..

[14] Foppa I.M., Krause P.J., Spielman A., Goethert H., Gern L., Brand B., Telford S.R. (2002). Entomologic and Serologic Evidence of Zoonotic Transmission of *Babesia Microti*, Eastern Switzerland. Emerg. Infect. Dis..

[15] Rar V.A., Epikhina T.I., Livanova N.N., Panov V.V. (2011). Genetic Diversity of *Babesia* in Ixodes Persulcatus and Small Mammals from North Ural and West Siberia, Russia. Parasitology.

[16] Hunfeld K.-P., Lambert A., Kampen H., Albert S., Epe C., Brade V., Tenter A.M. (2002). Seroprevalence of *Babesia* Infections in Humans Exposed to Ticks in Midwestern Germany. J. Clin. Microbiol..

[17] Wilhelmsson P., Lovmar M., Krogfelt K.A., Nielsen H.V., Forsberg P., Lindgren P.E. (2020). Clinical/Serological Outcome in Humans Bitten by *Babesia* Species Positive Ixodes Ricinus Ticks in Sweden and on the Aland Islands. Ticks Tick Borne Dis..

[18] Hunfeld K.-P., Brade V. (2004). Zoonotic *Babesia*: Possibly Emerging Pathogens to Be Considered for Tick-Infested Humans in Central Europe. Int. J. Med. Microbiol. Suppl..

[19] Zahler M., Rinder H., Schein E., Gothe R. (2000). Detection of a New Pathogenic *Babesia Microti*-like Species in Dogs. Vet. Parasitol..

[20] Baneth G., Florin-Christensen M., Cardoso L., Schnittger L. (2015). Reclassification of Theileria Annae as *Babesia* Vulpes Sp. Nov. Parasites Vectors.

[21] Baneth G., Cardoso L., Brilhante-Simões P., Schnittger L. (2019). Establishment of *Babesia* Vulpes n. Sp. (Apicomplexa: Babesiidae), a Piroplasmid Species Pathogenic for Domestic Dogs. Parasites Vectors.

[22] Camacho A.T., Pallas E., Gestal J.J., Guitián F.J., Olmeda A.S., Telford S.R., Spielman A. (2003). Ixodes Hexagonus Is the Main Candidate as Vector of Theileria Annae in Northwest Spain. Vet. Parasitol..

[23] Hodžić A., Zörer J., Duscher G.G. (2017). Dermacentor Reticulatus, a Putative Vector of *Babesia* Cf. *Microti* (Syn. Theileria Annae) Piroplasm. Parasitol. Res..

[24] Yeagley T.J., Reichard M.V., Hempstead J.E., Allen K.E., Parsons L.M., White M.A., Little S.E., Meinkoth J.H. (2009). Detection of *Babesia Gibsoni* and the Canine Small *Babesia* ‘Spanish Isolate’ in Blood Samples Obtained from Dogs Confiscated from Dog Fighting Operations. J. Am. Vet. Med. Assoc..

[25] Clark K., Savick K., Butler J. (2012). *Babesia Microti* in Rodents and Raccoons from Northeast Florida. J. Parasitol..

[26] Garrett K.B., Hernandez S.M., Balsamo G., Barron H., Beasley J.C., Brown J.D., Cloherty E., Farid H., Gabriel M., Groves B. (2019). Prevalence, Distribution, and Diversity of Cryptic Piroplasm Infections in Raccoons from Selected Areas of the United States and Canada. Int. J. Parasitol. Parasites Wildl..

[27] Modarelli J.J., Westrich B.J., Milholland M., Tietjen M., Castro-Arellano I., Medina R.F., Esteve-Gasent M.D. (2020). Prevalence of Protozoan Parasites in Small and Medium Mammals in Texas, USA. Int. J. Parasitol. Parasites Wildl..

[28] Holbrook A.A., Frerichs W.M. (1970). *Babesia* Mephitis Sp. n. (Protozoa: Piroplasmida), a Hematozoan Parasite of the Striped Skunk, Mephitis Mephitis. J. Parasitol..

[29] Goethert H.K., Cook J.A., Lance E.W., Telford S.R. (2006). Fay and Rausch 1969 Revisited: *Babesia Microti* in Alaskan Small Mammals. J. Parasitol..

[30] Goethert H.K., Lubelcyzk C., LaCombe E., Holman M., Rand P., Smith R.P., Telford S.R. (2003). Enzootic *Babesia Microti* in Maine. J. Parasitol..

[31] Bown K.J., Lambin X., Telford G.R., Ogden N.H., Telfer S., Woldehiwet Z., Birtles R.J. (2008). Relative Importance of *Ixodes Ricinus* and *Ixodes Trianguliceps* as Vectors for *Anaplasma Phagocytophilum* and *Babesia Microti* in Field Vole (Microtus Agrestis) Populations. Appl. Environ. Microbiol..

[32] Rar V., Yakimenko V., Makenov M., Tikunov A., Epikhina T., Tancev A., Bobrova O., Tikunova N. (2016). High Prevalence of *Babesia Microti* “Munich” Type in Small Mammals from an Ixodes Persulcatus/Ixodes Trianguliceps Sympatric Area in the Omsk Region, Russia. Parasitol. Res..

[33] Pieniążek N., Sawczuk M., Skotarczak B. (2006). Molecular Identification of *Babesia* Parasities Isolated from Ixodes Ricinus Ticks Collected in Northwestern Poland. Parasitology.

[34] Siński E., Bajer A., Welc R., Pawełczyk A., Ogrzewalska M., Behnke J.M. (2006). *Babesia Microti*: Prevalence in Wild Rodents and Ixodes Ricinus Ticks from the Mazury Lakes District of North-Eastern Poland. Int. J. Med. Microbiol..

[35] Welc-Falęciak R., Bajer A., Paziewska-Harris A., Baumann-Popczyk A., Siński E. (2012). Diversity of *Babesia* in *Ixodes Ricinus* Ticks in Poland. Adv. Med. Sci..

[36] Chen X.-R., Ye L.I., Fan J.-W., Li C., Tang F., Liu W., Ren L.-Z., Bai J.-Y. (2017). Detection of Kobe-Type and Otsu-Type *Babesia Microti* in Wild Rodents in China’s Yunnan Province. Epidemiol. Infect..

[37] Zamoto-Niikura A., Tsuji M., Qiang W., Nakao M., Hirata H., Ishihara C. (2012). Detection of Two Zoonotic *Babesia Microti* Lineages, the Hobetsu and U.S. Lineages, in Two Sympatric Tick Species, Ixodes Ovatus and Ixodes Persulcatus, Respectively, in Japan. Appl. Environ. Microbiol..

[38] Saito-Ito A., Takada N., Ishiguro F., Fujita H., Yano Y., Ma X.-H., Chen E.-R. (2008). Detection of Kobe-Type *Babesia Microti* Associated with Japanese Human Babesiosis in Field Rodents in Central Taiwan and Southeastern Mainland China. Parasitology.

[39] Saito-Ito A., Kasahara M., Kasai M., Dantrakool A., Kawai A., Fujita H., Yano Y., Kawabata H., Takada N. (2007). Survey of *Babesia Microti* Infection in Field Rodents in Japan: Records of the Kobe-Type in New Foci and Findings of a New Type Related to the Otsu-Type. Microbiol. Immunol..

[40] Tsuji M., Wei Q., Zamoto A., Morita C., Arai S., Shiota T., Fujimagari M., Itagaki A., Fujita H., Ishihara C. (2001). Human Babesiosis in Japan: Epizootiologic Survey of Rodent Reservoir and Isolation of New Type of *Babesia Microti*-Like Parasite. J. Clin. Microbiol..

[41] Bosman A.-M., Penzhorn B.L., Brayton K.A., Schoeman T., Oosthuizen M.C. (2019). A Novel *Babesia* Sp. Associated with Clinical Signs of Babesiosis in Domestic Cats in South Africa. Parasites Vectors.

[42] Penzhorn B.L., Oosthuizen M.C. (2020). *Babesia* Species of Domestic Cats: Molecular Characterization Has Opened Pandora’s Box. Front. Vet. Sci..

[43] Tsuji M., Zamoto A., Kawabuchi T., Kataoka T., Nakajima R., Asakawa M., Ishihara C. (2006). *Babesia Microti*-Like Parasites Detected in Eurasian Red Squirrels (*Sciurus Vulgaris OriEnt.is*) in Hokkaido, Japan. J. Vet. Med. Sci..

[44] Voorberg-vd Wel A., Kocken C.H.M., Zeeman A.-M., Thomas A.W. (2008). Detection of New *Babesia Microti*-like Parasites in a Rhesus Monkey (Macaca Mulatta) with a Suppressed Plasmodium Cynomolgi Infection. Am. J. Trop. Med. Hyg..

[45] Coles A. (1914). Blood Parasites Found in Mammals, Birds and Fishes in England. Parasitology.

[46] Franca C. (1908). Sur Une Piroplasme Nouvelle Chez Une Mangouste. Bull. Soc. Pathol. Exot..

[47] Anderson J.F., Johnson R.C., Magnarelli L.A., Hyde F.W., Myers J.E. (1986). Peromyscus Leucopus and Microtus Pennsylvanicus Simultaneously Infected with Borrelia Burgdorferi and *Babesia Microti*. J. Clin. Microbiol..

[48] Anderson J.F., Magnarelli L.A., Kurz J. (1979). Intraerythrocytic Parasites in Rodent Populations of Connecticut: *Babesia* and *Grahamella Species*. J. Parasitol..

[49] Kjemtrup A.M., Wainwright K., Miller M., Penzhorn B.L., Carreno R.A. (2006). *Babesia* Conradae, Sp. Nov., a Small Canine *Babesia* Identified in California. Vet. Parasitol..

[50] Conrad P.A., Kjemtrup A.M., Carreno R.A., Thomford J., Wainwright K., Eberhard M., Quick R., Telford III S.R., Herwaldt B.L. (2006). Description of *Babesia* Duncani n.Sp. (Apicomplexa: Babesiidae) from Humans and Its Differentiation from Other Piroplasms. Int. J. Parasitol..

[51] Armstrong P.M., Katavolos P., Caporale D.A., Smith R.P., Spielman A., Telford S.R. (1998). Diversity of *Babesia* Infecting Deer Ticks (*Ixodes Dammini*). Am. J. Trop. Med. Hyg..

